# Editorial: The Well-being of International Migrants in Rural Areas: Bridging the Migration-Development Nexus

**DOI:** 10.3389/fsoc.2022.870810

**Published:** 2022-04-15

**Authors:** Philomena de Lima, Belinda Leach, David Radford, Seema Arora-Jonsson

**Affiliations:** ^1^University of the Highlands and Islands-Inverness College, Scotland, United Kingdom; ^2^University of Guelph, Guelph, ON, Canada; ^3^University of South Australia, Adelaide, SA, Australia; ^4^Swedish University of Agricultural Sciences, Uppsala, Sweden

**Keywords:** migrants, refugees, rural, development, well-being, transnationalism, rural care

## Introduction

The presence of international migrants, asylum seekers and refugees in rural regions of Europe, North America and Australia has received growing attention as a means of facilitating development and the sustainability of rural regions and communities. Interventions to attract, recruit and retain international labor migrants and concerns about shortages of labor and skills in the context of aging populations and high levels of outmigration of the young and economically active in rural areas, have received increased research attention (Garela et al., 2018; de Lima and Carvajal, 2019; Haugen, 2019).

The debate and evidence on the contribution of migration to economic and social development in rural regions and elsewhere has evolved over time, and is ambivalent, messy and uncertain (Bastia, and Skeldon, 2021). As the articles in this issue reveal, migration to rural areas is imbricated in the dynamics of geopolitics and power hierarchies and changing national socio-cultural, economic and political circumstances. This development-migration nexus provides an important lens to highlight the multilayered relationship between the two, giving rise to both positive and negative impacts (Raghuram, 2009, 2020). The privileging of receiving countries' perspectives and instrumental arguments for recruiting transnational migrants are prominent in the academic and policy literature in rural regions of Europe, North America and Australia. National and EU policy discourses and policies on rural development often privilege notions of “unchanging” white rural areas which can counter policies on migration and “integration,” thus undermining substantive “integration” efforts in relation to migrants in receiving communities (Arora-Jonsson, 2017). With few exceptions (e.g. Preibisch and Hennebry, 2011; Preibisch, 2012; Bolokan, 2020; Dabrowska-Miciula and de Lima, 2020) there is a dearth of theoretical and empirical literature exploring migrants' wellbeing and their human rights within rural development discourses, a reflection of the presence of disciplinary silos in relation to migration, “development” including rural development, and wellbeing.

The overall aim of this special issue is to explore, illuminate and develop a more nuanced understanding of the relationship between migration, rural development, and the wellbeing of migrants, drawing on multiple perspectives, experiences, and methods in different national contexts. Taken together the papers demonstrate the importance of drawing on diverse theoretical perspectives and methodological approaches and of challenging persistent binaries (e.g. internal and international migration; sending and receiving countries) as well as exploring the experiences of different categories of migrants including refugees. The special issue also highlights a plurality of issues and experiences that form part of wider discourses associated with migration and development in rural areas, such as the economic contribution of migrants to host and home regions, the exploitation of temporary migrant workers in agriculture (and increasingly in other sectors as well) and the broad challenges of formulating ethical and practical policies. It also draws attention to novel areas of research inquiry. Here, we highlight a selection of these relatively underexplored issues discussed by the contributors which merit further discussion.

## Navigating Migrant Life in Rural Communities

The ways in which migrants become a part of local communities operate through a range of “domains” that include employment, housing, education and health, but also rights and citizenship, safety and stability, and language and cultural knowledge (Ager and Strong, 2008). It is clear in the papers by Boese and Moran, Bryan, Dauphinais, et al., Herslund and Paulgaard, and Sireni et al. that migrant wellbeing and their contributions to local communities are closely linked to positive social connections between migrants and local residents, as well as the context in which migration occurs and migrant aspirations and their imaginings of “rural places” (Papadopoulos and Fratsea). For example, Glorius et al. draw on acculturation literature to argue that a pre-requisite for successful integration and wellbeing for migrants in the long term has much to do with the quality and quantity of migrants' social contacts, which they suggest is shaped by the particular socio-spatial characteristics (e.g. limited cultural diversity and stereotyping) of rural communities. Experiences of social acceptance through positive contact support a positive integration trajectory leading to stronger development outcomes for the rural localities themselves, whereas negative contact can lead to reduced relational engagement.

In the prevailing discourses on the migration-development nexus, transnational migrants are often “portrayed as heroic agents of development” (Glick Schiller, 2020), used to support arguments about “inclusive growth” and development leading to what is promoted as “win-win” for nation states in destination and countries of origin. In contrast, considering the nexus between migration, development, and wellbeing, Boese and Moran draw attention to migrants' own development needs, rather than only focusing on the economic development of rural communities. They pose the question: what kind of development benefits migrants? In a parallel approach Dauphinais, et al., explore the role of mediating organizations, particularly employers, on the wellbeing and sense of acceptance and integration of international migrants in rural communities. Their research indicates that migrant dissatisfaction with living in rural Canada increases over time. They link this dissatisfaction to migrants' negative experiences of employment and the government's neo-liberal policies which focus on immigration as an economic endeavor rather than including a humanitarian element that focuses on migrants' wellbeing.

## Material and Physical Aspects of Rural Areas

Herslund and Paulgaard's contribution draws on the concept of “phenomenology of practices” (Simonsen, 2007), which emphasizes the bodily and sensory experiences of daily life that can spur feelings of “orientation” or “disorientation” in rural areas. Building on Ingold's work (Ingold, 2010) on the importance of weather, they explore the ways in which rural spaces—characterized by remoteness, visually unfamiliar landscapes, darkness, cold and windy climate and sparse populations—shape bodily experiences and affective relationships of migrant bodies to that of others. The disorientation experienced by migrants because of factors associated with specific rural locations is countered by reorientating their activities through an emphasis on prioritizing relationships with fellow refugees and meaningful voluntary activities that achieve change.

Research in Europe (Askins, 2009; Arora-Jonsson and Ågren, 2019) has indicated how conceptualizations of nature and the environment are crucial in migrant-local relations in rural areas, especially as rural environments are imbricated in defining selective national identities/ethnicities as “white” spaces, which exclude migrants from these spaces or evoke feelings of being in the wrong place (see also Agyeman, 1990). Bolokan's emphasis on the importance of understanding migrants' engagement in the agricultural sector, encompassing “caring for and with humans, animals, plants and the soil” makes an important contribution to understanding the entanglements of migration in environmental relations. The customary focus on the “integration” of migrants in rural communities has paid limited attention to the lived and bodily experiences (including the visibility of different bodies) of migrants that is crucial for the wellbeing of themselves and the communities in which they settle, as well as for rural development.

## A Transnational Approach to Wellbeing and Development Beyond Destination Countries

Scholarship and policy makers on migrants to rural areas continue to be locked into a methodological national framework by focusing attention on movers and their integration into new communities. There is far less consideration of their continuing relationships with those left behind and the impact of these shifting relationships on households and communities in the countries and places of origin. This gap persists despite the important theorizing on transnational ties (Glick Schiller, and Faist, 2010; Çaglar and Glick Schiller, 2018) and on translocality (Hedberg and do Carmo, 2012; Greiner and Sakdapolrak, 2013). Both have significant implications for the relational understanding of place—rural and urban—facilitated by mobilities as well as the relationships between differently mobile people (Brickell, 2011). Papadopoulos and Fratsea examine how mobilities differentially benefit the development and wellbeing of rural areas and of locals (non-movers) and migrants (movers) in rural areas. They argue for the need to strengthen and support the role of internal and international migrants as positive agents of change for rural areas. This highlights the importance of further research that moves away from binary conceptualizations of place as well as of bounded categorizations of migrants prevalent in migration and rural development research to enhance understandings of how places can be mobilized and will change for all residents—movers and non-movers.

In a similar vein, Sireni et al. focus on North Karelia, a rural region close to the Finnish-Russian border. They examine the agency of Russian immigrant women in contributing to the resilience and wellbeing of the entire region, which they argue is invisible in public discourses. Analyzing regional newspaper accounts and interviews with Russian female immigrants to North Karelia, they find narrow instrumental views of migrants as addressing labor shortages. Their research highlights the women's own emphasis on the roles they play as “agents of development” by mobilizing their border proximity, translocal connections and ethno-cultural capital as resources to facilitate their engagement as contributors to the vitality of both sides of the border.

## Unequal Relations: Global, Regional, Rural and Urban

Labor migration in rural areas, especially forms of temporary labor, is rooted in unequal economic and political relations: globally between countries exporting labor and the destination countries, between rural and urban areas within countries and internally in receiving societies where exploitation is endemic (Preibisch, 2012; Beatson et al., 2017). Bolokan argues for a wholistic approach in understanding Moldovan rural care chains in the context of the agricultural sector. Framing her paper within a critical political economy perspective, she draws on a “decolonial life course” approach that situates people's life courses within local and global power relations that reveal colonial continuities. She follows the movement of migrants between countries to explore the consequences of their “hypermobility” for the places, relationships, households, and communities of those who have migrated and those who are left behind. Bolokan argues that rural care chains are embedded in global networks which have both local place and personal consequences in countries of origin, that are not always positive for individuals and households. This is an area of research that has been generally underexplored in rural migration literature.

Bryan also addresses the underlying inequality of global labor regimes that are supported by government actions of both sending and receiving nations. Migrant workers are often caught in between and are left to navigate their own sense of wellbeing (emotional, physical, mental, material) within the precarity of their temporary migration status while at the same time aiding regional or national development goals. Bryan argues that migrant “self-management” of both their personal and non-migrant family wellbeing, often neglected in research, can end in the relative security of permanent residency but does not take away from the precarity of the process that largely benefits employers and local development while reinforcing the “individualization of neo-liberal wellness”.

## Decentring the “Here and There” Approach

The “here” and “there” in the lives of migrants applies to those who move internally as much as internationally, an area with potential for further consideration. This is raised by Murray et al. who give particular attention to local place, household and community effects by focusing on inter-provincial migration in Canada. They point to the difficulties faced by women whose partners leave to work elsewhere in Canada, linked to the dynamics of rural communities where everyone knows each other, where people are judged whether they leave or stay, and migrant partners assume that life in the “home” community is easier for the person who does not move. Murray et al. suggest a kind of de-integration of people, a process of growing exclusion of those perceived to be less connected to home communities because they return only intermittently or because their focus is seen to be on family members living away. The academic and policy binary distinctions between internal and international migrants situated against the lived practices of mobile workers across and within national and regional borders contribute to a complex spatial politics relatively unexplored in migration scholarship (Vullnetari, 2020).

## Possibilities for Future Research

The breadth of papers in this special issue covers a wide range of issues in relation to wellbeing and development in rural communities. The papers also point to areas that require further scholarly engagement. This includes explicit critical engagement with “rural development” and theories of development in relation to migration, in particular how rural development is conceptualized, applied and measured. The assumptions underlying the concept/terms in relation to wellbeing and development used in the papers in this special issue call for a deeper exploration that would allow for a better understanding of the actual contribution of migration to rural development, which remains unclear.

A number of contributions departed from community-based studies, vital for understanding the migration-development and wellbeing nexus. However, a limitation of small scale, short term/one off projects makes it difficult to address issues such as development outcomes and wellbeing which need longer time frames. The contributions of Bolokan, Murray et al. and Papadopoulos and Fratsea highlight a potential for scholarship of internal and international migration across different scales and groups.

There is a need for approaches that enhance our understanding of the specificities related to internal and international migration embedded as they are within the global political economy and migration system (including its governance), and which are also simultaneously shaped by their respective spatialities, histories and geo-politics. Bolokan's extension of the concept of care in agriculture to encompass animals, plants and soil, the consequences of migration for sending societies and the utilization of life course decolonial methods plugs a potential gap in migration scholarship which merits further consideration. This calls for research that engages in longitudinal and/or broader research methodologies such as in-depth, long term “whole community” studies that show how places, people (migrants, long term residents, etc.) and communities change over time. Another relatively unexplored area for research on the diversity of migrants to rural areas could compare and contrast their lived experiences in relation to wellbeing and development that takes account of intersectional differences such as gender, education, ethnicity /race, social class and so on, and importantly how the material environment and conceptions of nature itself are a part of migration experiences.

Lastly, a major contribution of several of the essays in this special issue has been to move beyond a “receiving” society bias, to focus on the full experiences of migrants, such as their transnational and multinational networks of family and others across their life course. Future research would do well to attend to these complex spatial and temporal dimensions of the migration experience as they address the nexus between international migration, wellbeing and development in rural communities.

## Author Contributions

All authors listed have made a substantial, direct, and intellectual contribution to the work and approved it for publication.

## Conflict of Interest

The authors declare that the research was conducted in the absence of any commercial or financial relationships that could be construed as a potential conflict of interest.

## Publisher's Note

All claims expressed in this article are solely those of the authors and do not necessarily represent those of their affiliated organizations, or those of the publisher, the editors and the reviewers. Any product that may be evaluated in this article, or claim that may be made by its manufacturer, is not guaranteed or endorsed by the publisher.
